# Musculoskeletal Stretching and Arterial Stiffness: Systematic Review and Meta-analysis of Acute and Longitudinal Interventions

**DOI:** 10.1007/s40279-026-02483-8

**Published:** 2026-07-26

**Authors:** Jeongok G. Logan, Aiden J. Chauntry, Hui Zhao, Sunbok Park, Lee Stoner

**Affiliations:** 1 School of Nursing, University of Virginia, Charlottesville, VA USA; 2 Department Exercise and Sport Science, University North Carolina at Chapel Hill, Chapel Hill, NC USA; 3 School of Nursing, James Madison University, Harrisonburg, VA USA; 4 Human Movement Science Curriculum, University of North Carolina at Chapel Hill, Chapel Hill, NC USA; 5 Department of Epidemiology, Gillings School of Global Public Health, University of North Carolina at Chapel Hill, Chapel Hill, NC USA; 6 Center for Health Promotion and Disease Prevention, University of North Carolina at Chapel Hill, Chapel Hill, NC USA; 7 Scienceye, Hillsborough, NC USA

## Abstract

**Background:**

Musculoskeletal stretching, a low-skill and accessible exercise, may help lower cardiovascular disease (CVD) risk by reducing arterial stiffness (AS). The most well-established measure of AS is pulse wave velocity (PWV).

**Objectives:**

To determine (1) the effects of acute and longitudinal musculoskeletal stretching interventions on PWV in adults; and (2) whether the pooled effect is moderated by stretching prescription (i.e., frequency, intensity, time, and type [FITT]), study design (e.g., supervision), study quality and risk of bias, and study outcome (e.g., specific PWV segment).

**Data Sources:**

Electronic databases (PubMed, Scopus, Embase, and Web of Science) were searched from 1974 to 2025, using the search terms: stretching AND (arterial stiffness OR pulse wave velocity OR PWV OR vascular stiffness). The reference lists of relevant studies and previous reviews were also screened.

**Study Selection:**

The eligibility criteria included (1) adults aged 18 years or older; (2) randomized controlled, nonrandomized controlled, or crossover; (3) PWV outcome(s) measured; (4) published in English; (5) published since 1974; and (6) published in peer-reviewed journals.

**Appraisal and Synthesis Methods:**

Initially, 242 studies were identified. After screening, six studies on acute stretching and six studies on longitudinal stretching were included. Effect sizes were calculated as standardized mean differences (SMD). Data were pooled using a 3-level model with restricted maximum likelihood estimation, and the magnitude of the effect was adjudicated as trivial (< 0.2), small (0.2), moderate (0.5), or large (0.8).

**Results:**

Acute stretching led to a small, nonsignificant reduction (beneficial) in PWV (SMD =  − 0.22, *p* = 0.258), whereas longitudinal stretching resulted in a large and statistically significant reduction in PWV (SMD =  − 1.02, *p* = 0.012). The pooled effect size was significantly greater (more beneficial) when each stretch was repeated three or more times (versus less than three times, SMD contrast =  − 1.46, *p* < 0.001). Larger (beneficial) pooled effect sizes were also observed when stretching was prescribed for 5–7 days/week (versus 3–4 days/week, SMD contrast =  − 1.05, *p* = 0.149), when stretching sessions lasted at least 30 min (versus less than 30 min, SMD contrast =  − 0.79, *p* = 0.169), and when sessions were supervised (versus unsupervised, SMD contrast =  − 1.11, *p* = 0.130); however, these differences did not reach statistical significance.

**Conclusions:**

A single bout of stretching resulted in a small acute decrease in PWV (SMD =  − 0.22, 95% CI − 0.62 to 0.17). There was a large decrease in PWV (SMD =  − 1.02, 95% CI − 1.79 to − 0.25) following a 4- to 12-week intervention. We recommend stretching each major muscle–tendon group three or more times. These findings may be used to help design a future intervention to test the efficacy and biological plausibility of stretching exercises to reduce arterial stiffness and possibly CVD risk.

**Supplementary Information:**

The online version contains supplementary material available at https://doi.org/10.1007/s40279-026-02483-8.

## Key Points

**Table:** 

This meta-analysis tested the effects of acute and longitudinal musculoskeletal stretching interventions on pulse wave velocity (PWV) in adults. We observed a small, nonsignificant decrease in PWV after a single stretching session, whereas a large, significant decrease occurred following 4–12 weeks of stretching intervention, with effects nearing those seen with aerobic exercise interventions.
Our data suggest that repeating stretches for major muscle–tendon groups three or more times may enhance arterial stiffness reduction. These findings may guide future trials to test the efficacy and biological plausibility of stretching for reducing arterial stiffness.

## Introduction

Cardiovascular disease (CVD) remains a leading cause of global mortality [1]. While moderate-to-vigorous intensity physical activity interventions are effective at reducing CVD risk [2, 3], long-term compliance is often low [4]. Musculoskeletal static stretching (hereafter stretching) is a viable alternative; it requires minimal skill and can be performed in most locations, making it accessible and sustainable for a wide range of populations [5, 6]. Studies have investigated the acute (single-session) and longitudinal (multi-session) effects of stretching on cardiovascular outcomes, including arterial stiffness (AS), but the findings have been mixed [7–9]. The mixed findings may be attributable, at least in part, to studies using small sample sizes, differences in study methodology, and variability in the stretching prescription. A systematic review is required to: (1) harmonize the literature; (2) determine the optimal study design and stretching prescription; and (3) identify gaps in knowledge that need to be filled to progress our understanding of the value of stretching interventions to mitigate CVD risk.

The value derived from meta-analytical findings depends on the quality of the selected intermediate outcome, i.e., an outcome on the causal pathway between the exposure/treatment variable (e.g., stretching) and the distal health outcome (e.g., CVD) [10]. AS refers to a reduced capacity for arteries to expand and contract in response to changes in blood pressure. Healthy, flexible arteries buffer the pulsatile flow of blood, but when arteries become stiff, this function is impaired, which can compromise overall vascular health and increase the risk of CVD [11, 12]. AS can be measured noninvasively using pulse wave velocity (PWV), or the velocity (m/s) at which the forward pressure wave propagates between a proximal and distal arterial segment. PWV is commonly used as a noninvasive measure of AS and serves as an established predictor of CVD risk [13]. Higher PWV values indicate stiffer arteries. Among different PWV measures, carotid-femoral PWV is considered the gold standard for assessing central (aortic) AS and is strongly associated with future cardiovascular events. For example, a prior meta-analysis reported that an increase of 1 m/s in aortic PWV is associated with a 14% elevated risk of future cardiovascular events [12]. Other measures, such as brachial-ankle PWV, or peripheral PWV are also used in research, especially in Asian countries, but these measures reflect both central and peripheral AS and have shown inconsistent associations with CVD risk [14].

A recent meta-analysis revealed a significant relationship between poor trunk flexibility and increased AS, particularly in older individuals [15, 16]. This finding highlights the need for targeted interventions to enhance flexibility, helping to mitigate age-related increases in AS and CVD risk. There is a biologically plausible relationship between stretching and AS [15]. AS is dependent on the functional and structural characteristics of a vessel [12, 13, 17, 18]. Functionally, AS is influenced by endothelial function [11, 19, 20]. Structurally, AS is dependent on the vessel wall’s extracellular matrix, including the content of elastin and collagen [11]. A single stretching bout may temporarily decrease AS through acute improvements in endothelial function, plausibly as a function of shear-stress mediated increases in nitric oxide (a key molecule regulating endothelial function) production [21, 22]. Repeated acute improvements in endothelial function, resulting from regular exposure to stretching (i.e., a longitudinal stretching intervention), may lead to sustained reductions in AS through extracellular matrix remodeling [23]. For these reasons, it is important to garner a better understanding of both the acute and longitudinal effects of stretching, separately, on PWV.

Two previous meta-analyses have investigated the effect of stretching intervention on AS [24, 25]. Thomas et al. pooled data from eight studies with a combined 216 participants and reported a large reduction in brachial-ankle PWV, (standardized mean difference [SMD] =  − 2.04), and a small-moderate reduction in carotid-femoral PWV (SMD =  − 0.46) [24]. Kato et al. pooled eight studies with a combined 213 participants and reported a large (SMD =  − 1.00) reduction in their pooled arterial stiffness outcome [25]. While the findings from these previous meta-analyses are important, there are some limitations [24, 25], including: (1) combining acute and longitudinal stretching interventions into one model; (2) pooling PWV and PWV-associated outcomes, including cardio-ankle vascular index (CAVI) and augmentation index—a measure of arterial wave reflection; and (3) not adequately accounting for multiple effects nested within a given study, thereby violating the assumption of observation independence. Additionally, Kato et al. focused exclusively on middle-aged and older adults [25], and both meta-analyses were restricted to studies published before January 2020 [24, 25]. A new systematic review and meta-analysis are needed for several reasons. First, the previous meta-analyses did not calculate the independent pooled effect of acute stretching on PWV. As alluded to in the prior paragraph, this information would aid in the identification of the optimal stretching stimulus. Second, the prior meta-analyses did not test whether the stretching prescription moderates the association between stretching and AS. There are guidelines for stretching exercises, last updated in 2021, but these guidelines were developed to improve flexibility, not CVD risk [26]. In alignment with the FITT (frequency, intensity, time, and type) principle, we do not know the optimal frequency (sessions/week), time (stretch hold time, repetitions, and min/session), or type (muscles–tendon groups stretched and passive versus active) of stretching intervention required to most effectively reduce AS. Additionally, it is essential to consider the importance of study design factors (e.g., whether the stretching intervention was supervised), and study outcomes (i.e., the AS measurement methods and PWV segment assessed) used. For the acute stretching studies, it is also important to confirm the duration (in minutes) between the last stretch and the measurement of PWV. Some studies have measured PWV immediately or within 10 min following the final stretch [7, 27–30]. The resultant PWV data may be artificial, possibly reflecting a function of heightened hemodynamic activity residual to the stretching exercise, or the postural change to a supine position for PWV assessment [31]. A comprehensive understanding of these moderating factors would inform the refinement of stretching protocols, guide the development of more effective stretching interventions, and contribute to updating policy guidelines for stretching practices to reduce CVD risk.

## Objectives

The primary aim of this study was to determine the effect of (1) acute and (2) longitudinal musculoskeletal stretching interventions (randomized controlled, nonrandomized controlled, crossover trials) on PWV in adults aged 18 years or older. A secondary aim was to determine whether the pooled effect was moderated by stretching prescription (i.e., FITT), study design (e.g., supervision), study quality and risk of bias, and study outcome (e.g., PWV segment).

## Methods

This systematic review was registered with the International Prospective Register of Systematic Reviews (PROSPERO, ID: CRD42024524199) and followed the Preferred Reporting Items for Systematic reviews and Meta-Analyses (PRISMA) reporting guidelines [32].

### Data Sources and Searches

Two authors (J. G. L. and A. J. C.) conducted searches across electronic databases (PubMed, Scopus, Embase, and Web of Science) in December 2025. The following search terms: stretching AND (arterial stiffness OR pulse wave velocity OR PWV OR vascular stiffness) were used. In addition, the reference lists of all identified articles and relevant reviews or editorials were also examined. The search was limited to human participants and English-language studies published from 1974 onward, when research on arterial stiffness and vascular biomarkers began to expand. A fully replicable version of a complete PubMed search strategy is provided in Supplementary Table 2.

### Article Selection

Two researchers (J. G. L. and A. J. C.) completed the study selection independently. Conflicts between reviewers were resolved through discussion or, when an agreement could not be reached, by a third author (L. S.). The term “article” is used synonymously with “study” (*u*), and *effect size* (*k*) is the unit included in the review. A given* u* may have resulted in more than one eligible *k* if separate independent samples (e.g., male and female) or experimental conditions (e.g., control versus condition 1 and control versus condition 2) were used within a given *u*. In such cases, data for each estimate were extracted separately. Duplicate studies and repeated publications from the same *u* (i.e., repeated use of the same dataset) were excluded. The titles and abstracts, and full texts of potentially eligible articles were screened, and studies were included in the review if they met the following criteria: (1) adults aged 18 years or older; (2) randomized controlled, nonrandomized controlled, or crossover; (3) PWV outcome(s) were measured; (4) published in English; (5) published since 1974; and (6) published in peer-reviewed journals. Studies were excluded if they were systematic reviews, meta-analyses, abstracts, letters to the editor, or animal studies. Among eligible studies, those with extractable or imputed data were included in the meta-analysis.

### Study Methodological Quality and Bias Assessments

Study quality was assessed by two researchers (J. G. L. and H. Z.) using the Critical Appraisal Skills Programme (CASP) checklist for randomized control trials (RCTs) [33], which includes 11 items related to the validity of study design, methodology assessment, and reporting and application of results. Each item was scored either 1 (yes) or 0 (no or can’t tell). The summed score ranges between 11 (highest quality) and 0 (lowest quality).

The risk of bias within studies was assessed by two researchers (J. G. L. and H. Z.) using the Cochrane risk-of-bias 2 (ROB-2) assessment tool [34]. ROB-2 includes items related to bias arising from randomization process, period and carryover effects, deviations from intended interventions, missing outcome data, measurement of the outcome, and selection of the reported result. Each effect size was assigned one of the following three aggregate scores: “low risk of bias,” “some concerns,” or “high risk of bias.”

### Data Extraction and Synthesis

Data extraction and synthesis were completed by two researchers (J. G. L. and A. J. C.). Once both authors had completed data extraction, their values were compared. If a given data point differed by < or > 5%, both authors rechecked their extraction. The final data set consisted of values averaged across authors. The following data were extracted and entered into a spreadsheet: (1) bibliographic information (author, publication year); (2) sample characteristics (e.g., age, sex, race, health condition, etc.); (3) details of intervention (country, setting, study design, time between last stretch and PWV measurement, stretch site, stretch symmetry, active/passive, holding time, relaxation time, number of repeats, sessions per week, durations, supervised or not, and control condition); and (4) pre- and postintervention outcome data (carotid-femoral, brachial-ankle, carotid-radial, brachial-femoral, femoral-ankle, heart-ankle PWV). For studies that reported *k* > 1, either across subgroups or within subjects, we applied a correction by adjusting the sample size (adjusted sample size = original sample size/*k*) [35].

Missing data were, in order of priority and depending on availability: (1) requested from the corresponding and senior author; (2) extracted from a figure; (3) estimated/imputed on the basis of methods from the Cochrane Handbook [35]; or (4) excluded. As a means of quality control, we imputed the data before contacting authors (if possible). Our estimate was then compared with the data provided by the corresponding/senior author. If the authors were unresponsive on the first attempt, two subsequent attempts were made, each 2 weeks apart. A study was excluded if the data could not be imputed and the authors did not respond to our contact attempts.

Data were extracted from figures using a data digitizing application (PlotDigitizer: https://plotdigitizer.com) [36]. To estimate the potential error introduced by extracting data from figures, each researcher initially extracted data from a training figure set. The training set comprised five figures, from articles published by our group, and 32 data points, for which the true data values were known. The figures included various graphical formats and variance estimates (i.e., standard deviation, standard error, and confidence intervals). Each researcher independently extracted data from each figure three times, closing and recalibrating the digitizer each time. To determine validity, the extracted data for each researcher were correlated (Pearson’s residual (*r*)) against the known values. Intra-rater reliability was then determined using a one-way mixed, absolute agreement, average-measures intra-class correlation coefficient (ICC) [37]. Following the completion of the training set, the same process was used to extract data for this meta-analysis. Before actual data extraction, the author had to achieve acceptable reliability (*r* ≥ 0.95) and agreement (ICC ≥ 0.90) [37]. If the data could not be extracted from a figure, the standard deviation (SD) was estimated using the: (1) standard error (SE) x √*n*; (2) 95% confidence interval (CI): (upper confidence interval [UCI] − lower confidence interval [LCI])/3.92; (3) 90% CI: (UCI − LCI)/3.29; (4) baseline SD if the postexposure SD was missing; or (5) SD from closest study if both the pre-exposure and postexposure SDs were missing [35].

### Data Analysis

A single author (L. S.) conducted the data analysis using RStudio (version 2024.09.01) within the R statistical environment (version 4.3.1) [38]. For each statistical procedure, we specified the R argument and package used as “(argument[package]).” The *α* level was set a priori at *α* = 0.05 for the pooled effect estimate and *α* = 0.10 for the moderator analysis and the *Q* test.

Two meta-analyses were completed to calculate the pooled effect of acute stretching interventions and longitudinal stretching interventions, respectively. The pooled effects are expressed as mean differences (MDs) and standardized mean differences (SMDs). The pooled SMDs were used to test the study objectives, considering that the different measures of PWV (e.g., carotid-femoral PWV versus femoral-ankle PWV) may not be directly comparable. The MDs were used to aid the interpretation of clinical significance. The magnitude of the pooled SMD was interpreted as trivial (< 0.2), small (0.2), moderate (0.5), or large (0.8) [39]. A 1 m/s change in PWV for the longitudinal interventions was considered clinically significant [12]. Corresponding 95% confidence intervals (95% CI) and prediction intervals were also calculated. The 95% CI reflects the precision of the observed effect, whereas the prediction reflects the dispersion of the true effect—i.e., the interval within which the effect size of a new study would be expected to fall [40, 41].

The data were fitted using a multivariate, 3-level model with restricted maximum likelihood estimation [42, 43]. To ensure full transparency, we also estimated the pooled effect using univariate fixed-effects and random-effects models (DerSimonian–Laird method) [44, 45]. The 3-level model accounted for *k* dependency when a given *u* reported more than one *k* [42, 43]. Variance (*τ*^2^) was calculated for between-study (level 3 *τ*^2^), within-study (level 2 *τ*^2^), and sampling (level 1 *τ*^2^). Full (3-level) and reduced (2-level and 1-level) model fit data were compared (anova[metafor]) using Akaike’s information criterion, corrected for small samples (AICc), and the log-likelihood ratio test. After running the 3-level model, we tested the following: (1) pooled estimate robustness; (2) heterogeneity; and (3) a priori moderators.

1. The robustness of the pooled SMD and 95% CI was confirmed using permutation tests (permutest[metafor]) [38]. Pearson’s residuals (residuals.rma[metafor]), hat values (hatvalues[metafor]), and Cook’s distances (cooks.distance[metafor]) were calculated to test for potential outliers or influential data, at *k* and *u* levels [46]. A Pearson’s residual (*r*^2^) greater than 3.84 (the *χ*^2^ (1) cutoff at *α* = 0.05) indicated a potential outlier. A hat or Cook’s distance value greater than 3 × (1/[*k* or *u*]) or 0.5, respectively, indicated potentially influential *k* or *u*. Leverage (*x* = hat, *y* = Pearson’s, size = Cook’s) and Baujat (*x* = Pearson’s *r*^*2*^, *y* = Cook’s) plots (ggplot[ggplot2]) were constructed to visually identify whether a *k* or *u* required closer inspection (e.g., for study quality, bias, small sample size, etc.). Publication bias was qualitatively examined by inspecting Begg’s funnel plot for asymmetry [47]. The fail-safe *N* was also calculated to estimate the number of unpublished null studies that would be needed to nullify the observed effect. Asymmetry was quantitatively tested using the regression intercept test, with the standard error of the observed outcomes specified as a moderator in the 3-level model.
2. Heterogeneity was assessed using Cochran’s *Q*-test [48] and Higgins’ *I*^2^ statistic [49]. The *I*^2^ statistic was used to adjudicate the heterogeneity as low (< 25%), moderate (25–75%), or high (> 75%). A greater than low heterogeneity (≥ 25%) suggests meaningful heterogeneity. A prediction interval for the pooled effect estimate was calculated if any amount of heterogeneity was detected (i.e., *τ*^2^ > 0) [40].
3. Potential moderators were specified independently. Owing to the small number of included *k*, the continuous moderator variables were all expressed as factors. The variance explained (pseudo *R*^2^) through moderator specification was estimated by calculating the percentage reduction in *τ*^2^ at each level (*u*, *k*, and sampling) of the model. Model fit was evaluated using AICc and the log-likelihood ratio test (anova[metafor]). The multicollinearity assumption was tested using VIF (vif.ram[metafor]), where 1 = not correlated, 1–5 = moderate, and > 5 = high.

### Certainty Assessment

The certainty of evidence for each outcome was assessed using the grading of recommendations assessment, development and evaluation (GRADE) approach. Evidence derived from randomized controlled trials was initially rated as high certainty. The certainty of evidence was then downgraded on the basis of predefined criteria, including risk of bias, inconsistency (unexplained heterogeneity), indirectness of evidence, imprecision (wide confidence intervals or small sample sizes), and potential publication bias. Where applicable, evidence could be rated up on the basis of factors such as large magnitude of effect, evidence of dose–response relationships, or if all plausible residual confounding would reduce a demonstrated effect. Disagreements in ratings were resolved through discussion among the review authors [50].

## Results

### Literature Search and Trial Selection

**Fig. 1:**
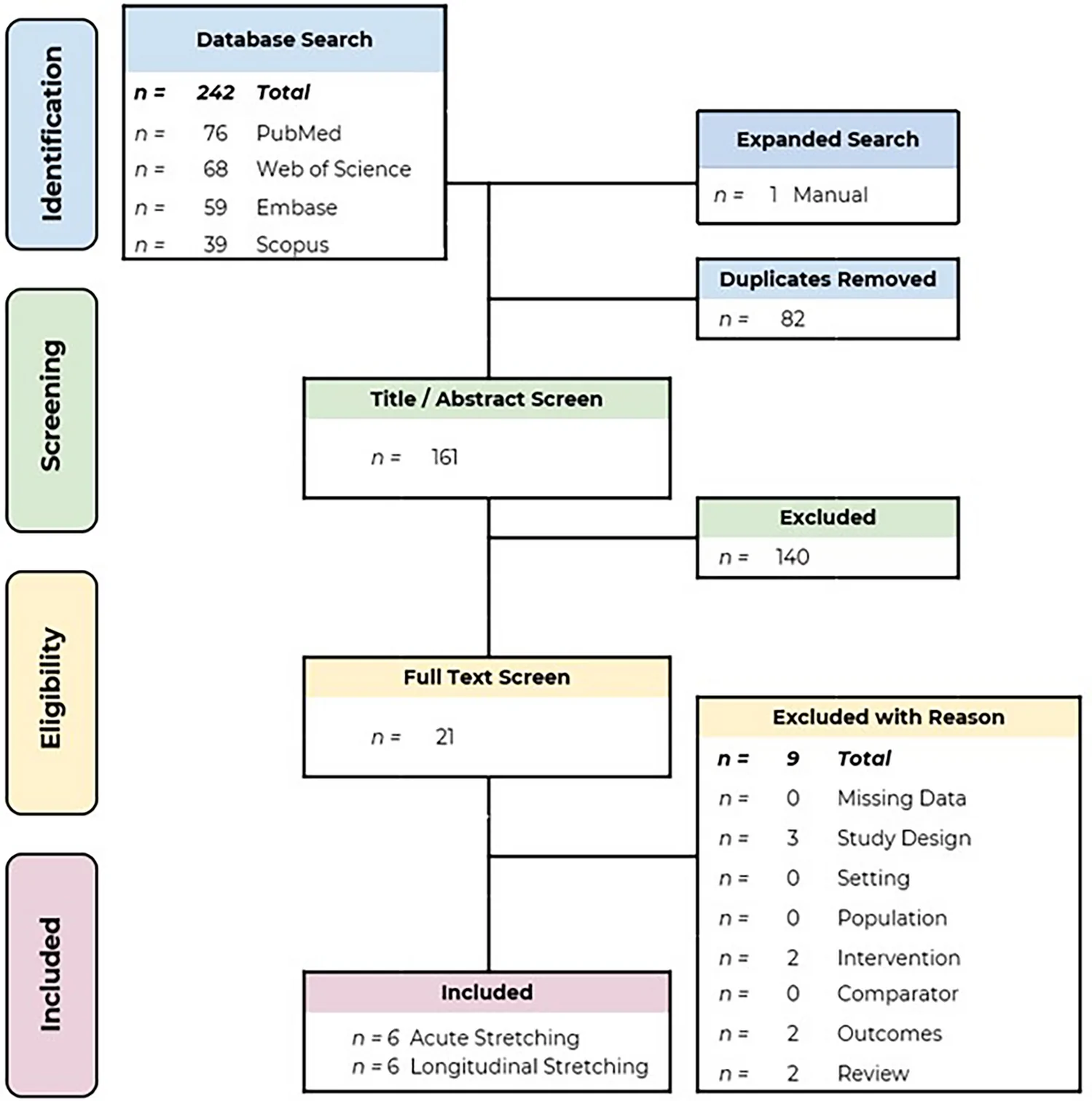
Flow diagram depicting study selection process

The literature searches for the acute- and longitudinal-stretching intervention studies were conducted concurrently. The search strategy and outcomes are outlined in Fig. 1. Initial database searches identified a total of 242 potentially eligible studies (*u*). One additional *u* was identified through an expanded search. Following the removal of duplicates, and title and abstract screening, 222 *u* were excluded. An additional 9 *u* were excluded after full-text screening for the following reasons: reviews (*n* = 2), wrong outcomes (*n* = 2), wrong intervention (*n* = 2), and wrong study design (*n* = 3). Of the 12 *u* remaining, 8 *u* had missing data. We were able to obtain from the authors or extract the data for all 8 *u*. For the acute stretching meta-analysis, 26 k were extracted from 6 *u* [7, 8, 27, 28, 51, 52], all of which were published in the past 7 years. For the longitudinal stretching meta-analysis, 17 k were extracted from 6 *u* [9, 29, 30, 53–55], all of which were published in the past 10 years. Below, we highlight key findings from the meta-analyses. Further details about each *k*/*u* can be found in Table 1 for the acute stretching studies and Table 2 for the longitudinal stretching studies.

**Table 1:** Characteristics of acute stretching studies

Study	*u*	*k*	Age (years)	Sex	*n*	Race	Healthy	Country	Setting	Outcome	Supervised	Control condition	Quality	Risk of bias
Higaki et al. [27]	1	1	23.1	Male	17	Asian	Yes	Japan	Community	BA	Yes	Unilateral	8	High
Higaki et al. [27]	1	2	23.1	Male	17	Asian	Yes	Japan	Community	BA	Yes	Unilateral	8	High
Higaki et al. [27]	1	3	23.1	Male	17	Asian	Yes	Japan	Community	BA	Yes	Unilateral	8	High
Higaki et al. [27]	1	4	23.1	Male	17	Asian	Yes	Japan	Community	BA	Yes	Unilateral	8	High
Higaki et al. [27]	1	5	23.1	Male	17	Asian	Yes	Japan	Community	FA	Yes	Unilateral	8	High
Higaki et al. [27]	1	6	23.1	Male	17	Asian	Yes	Japan	Community	FA	Yes	Unilateral	8	High
Higaki et al. [27]	1	7	23.1	Male	17	Asian	Yes	Japan	Community	FA	Yes	Unilateral	8	High
Higaki et al. [27]	1	8	23.1	Male	17	Asian	Yes	Japan	Community	FA	Yes	Unilateral	8	High
Ikebe et al. [7]	2	9	21.3	Male	12	Asian	Yes	Japan	University	CF	Yes	Timed	8	Some concerns
Ikebe et al. [7]	2	10	21.3	Male	12	Asian	Yes	Japan	University	BA	Yes	Timed	8	Some concerns
Ikebe et al. [7]	2	11	21.3	Male	12	Asian	Yes	Japan	University	HA	Yes	Timed	8	Some concerns
Ikebe et al. [7]	2	12	21.3	Male	7	Asian	Yes	Japan	University	CF	Yes	Timed	8	Some concerns
Ikebe et al. [7]	2	13	21.3	Male	12	Asian	Yes	Japan	University	CF	Yes	Timed	8	Some concerns
Ikebe et al. [51]	3	14	23.7	Both	17	Asian	Yes	Japan	University	HA	Yes	Timed	8	Some concerns
Ikebe et al. [51]	3	15	23.7	Both	17	Asian	Yes	Japan	University	HA	Yes	Timed	8	Some concerns
Ikebe et al. [51]	3	16	23.7	Both	17	Asian	Yes	Japan	University	HA	Yes	Timed	8	Some concerns
West et al. [28]	4	18	72	Female	14	NR	NR	USA	Community	CF	Yes	Timed	10	Some concerns
West et al. [28]	4	17	72	Male	14	NR	NR	USA	Community	CF	Yes	Timed	10	Some concerns
Yamato et al. [8]	5	19	20.9	Male	25	Asian	Yes	Japan	Community	FA	Yes	Unilateral	8	High
Yamato et al. [8]	5	20	20.9	Male	25	Asian	Yes	Japan	Community	FA	Yes	Unilateral	8	High
Yamato et al. [8]	5	21	20.9	Male	25	Asian	Yes	Japan	Community	FA	Yes	Unilateral	8	High
Yamato et al. [8]	5	22	20.9	Male	25	Asian	Yes	Japan	Community	BA	Yes	Unilateral	8	High
Yamato et al. [8]	5	23	20.9	Male	25	Asian	Yes	Japan	Community	BA	Yes	Unilateral	8	High
Yamato et al. [8]	5	24	20.9	Male	25	Asian	Yes	Japan	Community	BA	Yes	Unilateral	8	High
Okamoto et al. [52]	6	25	22	Male	12	Asian	Yes	Japan	University	BA	Yes	Timed	9	Some concerns
Okamoto et al. [52]	6	26	22	Male	12	Asian	Yes	Japan	University	BA	Yes	Timed	9	Some concerns

*u* study, *k* effect size, *PWV* pulse wave velocity, *CF* carotid-femoral, *CR* carotid-radial, *BA* brachial-ankle, *FA* femoral-ankle, *HA* heart-ankle, *NR* not reported

**Table 2:** Characteristics of longitudinal stretching studies

Study	*u*	*k*	Age (years)	Sex	*n*	Race	Healthy	Country	Setting	PWV	Supervised	Control condition	Quality	Risk of bias
Bisconti et al. [53]	1	1	23.0	Male	13	White	Yes	Italy	University	CF	Yes	Usual care	7	Some concerns
Bisconti et al. [53]	1	2	23.0	Male	13	White	Yes	Italy	University	CR	Yes	Usual care	7	Some concerns
Bisconti et al. [53]	1	3	23.0	Male	13	White	Yes	Italy	University	CF	Yes	Usual care	7	Some concerns
Bisconti et al. [53]	1	4	23.0	Male	13	White	Yes	Italy	University	CR	Yes	Usual care	7	Some concerns
Bisconti et al. [53]	1	5	23.0	Female	13	White	Yes	Italy	University	CF	Yes	Usual care	7	Some concerns
Bisconti et al. [53]	1	6	23.0	Female	13	White	Yes	Italy	University	CR	Yes	Usual care	7	Some concerns
Bisconti et al. [53]	1	7	23.0	Female	12	White	Yes	Italy	University	CF	Yes	Usual care	7	Some concerns
Bisconti et al. [53]	1	8	23.0	Female	12	White	Yes	Italy	University	CR	Yes	Usual care	7	Some concerns
Boonpim et al. [29]	2	9	55.0	Female	40	NR	Post menopausal	Thailand	Community	BA	Yes	Usual care	5	Some concerns
Higaki et al. [9]	3	10	65.3	Female	14	Asian	Post menopausal	Japan	Community	CF	No	Usual care	5	Some concerns
Higaki et al. [9]	3	11	65.3	Female	14	Asian	Post menopausal	Japan	Community	BA	No	Usual care	5	Some concerns
Higaki et al. [9]	3	12	65.3	Female	14	Asian	Post menopausal	Japan	Community	FA	No	Usual care	5	Some concerns
Nishiwaki et al. [55]	4	13	43.0	Male	16	Asian	Yes	Japan	University	BA	Yes	Usual care	5	Some concerns
Shinno et al. [30]	5	14	47.4	Female	22	NR	Yes	Japan	NR	BA	No	Timed	5	Some concerns
Wong et al. [54]	6	15	57.0	Female	28	Mixed	Obese and post menopausal	USA	Community	CF	Yes	Usual care	7	Some concerns
Wong et al. [54]	6	16	57.0	Female	28	Mixed	Obese and post menopausal	USA	Community	FA	Yes	Usual care	7	Some concerns
Wong et al. [54]	6	17	57.0	Female	28	Mixed	Obese and post menopausal	USA	Community	BA	Yes	Usual care	7	Some concerns

*u* study, *k* effect size, *PWV* pulse wave velocity, *CF* carotid-femoral PWV, *CR* carotid-radial PWV, *BA* brachial-ankle PWV, *FA* femoral-ankle PWV, *NR* not reported

### Study Participants and Setting

#### Acute Stretching Studies

The total *n* for acute stretching studies was 110, with a median of 17 per *u*. The mean sample age ranged from 21 years [8] to 72 years [28], with a median of 22.6 years. The reported sex was all male for 22 k [7, 8, 27, 28, 52], all female for 1 k [28], and mixed sex for 3 k [51]. Race/ethnicity was reported for 24 k*,* all of which were Asian and conducted in Japan [7, 8, 27, 51, 52]. The remaining *2 k* (1* u*) were conducted in the USA and did not report race/ethnicity [28].

#### Longitudinal Stretching Studies

The total *n* for longitudinal stretching studies was 159 with a median of 25 per *u*. The mean sample age ranged from 23 [53] to 65 years [9], with a median age of 43 years. The reported sex was all female for 12 k [9, 29, 30, 53, 54], and all male for 5 k [53, 55]. Race/ethnicity was reported in 15 k [9, 53–55], including White for 8* k* (1 *u*) [53], Asian for 4 k (2 *u*) [9, 55], and mixed (White, African American, and Hispanic) for 3* k* (1 *u*) [54]. A total of 3 *u* were conducted in Japan [9, 30, 55] and one each in the USA [54], Italy [53], and Thailand [29].

### Study Design and Interventions

#### Acute Stretching Studies

The control condition was the contralateral limb for 14 k [8, 27] and a timed control for 12 k [7, 28, 51, 52]. The measured outcome was brachial-ankle PWV for 10 k [7, 8, 27, 52], femoral-ankle PWV for 7 k [8, 27], carotid-femoral PWV for 5 k [7, 28], and heart-ankle PWV for 4 k [7, 51]. The timing of PWV assessment following the last stretching exercise ranged from immediately poststretch to 30 min poststretch [8, 51, 52], with a median time of 5 min. The stretching prescription varied between *k*. Regarding frequency, a single session was used for all *k*. In terms of intensity, stretching was performed to the point of mild discomfort for all *k*. As for the duration (time), each stretching session lasted 3.5 [27] to 30 [7, 28] min, with a median of 5 min. Each stretch was repeated 2 [51] or 6 [7, 8, 27] times. For moderation analysis, duration per session was dichotomized into low (1–15 min/session) or high (16–30 min/session), and repetitions per stretch into low (< 3 repetitions) or high (≥ 3 repetitions). With respect to type, 16 k prescribed only lower-body stretching exercises [7, 8, 27], 8 k only upper-body exercises, either trunk [7, 28] or cervical stretching [51], and 2 k whole body stretching [52]

#### Longitudinal Stretching Studies

Regarding study design, 5 *u* employed a randomized controlled design [9, 29, 30, 53, 54] and 1 *u* [55] used a nonrandomized controlled design. A total of 16 k used usual care as the control condition [9, 29, 53, 54] and 1 k offered optional stretching seminars [30]. The measured outcome was carotid-femoral PWV for 6 k [9, 53, 54], brachial-ankle PWV for 5 k [9, 29, 30, 54, 55], carotid-radial PWV for 4 k [53], and femoral-ankle PWV for 2 k [9, 54]. The stretching prescription varied among *k*. Regarding frequency, stretching was performed between three [54] and seven sessions/week [30], with a median of five sessions/week. In terms of intensity, stretches were performed to the point of mild discomfort for all *k*. For the duration (time), each stretching session lasted 13 [9] to 50 min [52], with a median value of 30 min, and each stretch was held for 20 [29] to 45 s [53], with a median of 30 s, and repeated two [9, 52] to five times [53], with a median of four repetitions. Intervention duration spanned from 4 [9] to 12 weeks [30, 53], with a median of 12 weeks, with the total stretching exposure time ranging from 3.6 [9] to 40 h [53], with a median of 20 h. With respect to type, 6 k tested whole body stretching [29, 30, 54, 55], while 11 k tested leg stretching [9, 53]. Between 4 [53] and 38 stretching exercises [54] were conducted per session, with a median value of 10 stretching exercises.

### Study Methodological Quality and Bias

#### Acute Stretching Studies

The methodological assessment of included trials is summarized in Table 1. The potential for within-study bias is summarized in Supplementary Table 3 and Supplementary Fig. 1. The quality of the included studies ranged from eight [7, 8, 27, 51] to ten [28], with a median quality score of eight. All 6 *u* had a score of seven (high quality) or above. Four studies had some concerns of bias [7, 28, 51, 52] and two studies had a high risk of bias [8, 27]. None of the studies, except 1 *u* [52], provided information about the blinding of the measurers, which raises some concerns. In two studies [8, 27], both the intervention and control were conducted on the same participants, with one leg being stretched while the other nonstretched leg served as a control. This design introduced potential carry-over effects, posing a high risk of bias, despite being an intentional aspect of the study. For moderation analysis, risk of bias was dichotomized to low (some risk) or high (high risk).

#### Longitudinal Stretching Studies

The methodological assessment of included trials is summarized in Table 2, with the potential for within-study bias outlined in Supplementary Table 4 and Supplementary Fig. 1. The quality of the included studies ranged from five [9, 29, 30, 55] to seven [53, 54], with a median quality score of six. All six studies had some concerns of bias [9, 29, 30, 53–55]. None of the studies provided information about the blinding of the measurer, nor did they sufficiently describe the process for concealing the allocation sequence. For moderation analysis, study quality was dichotomized to low (< 7) or high (≥ 7).

### Synthesis of the Results

#### Acute Stretching Studies

**Fig. 2:**
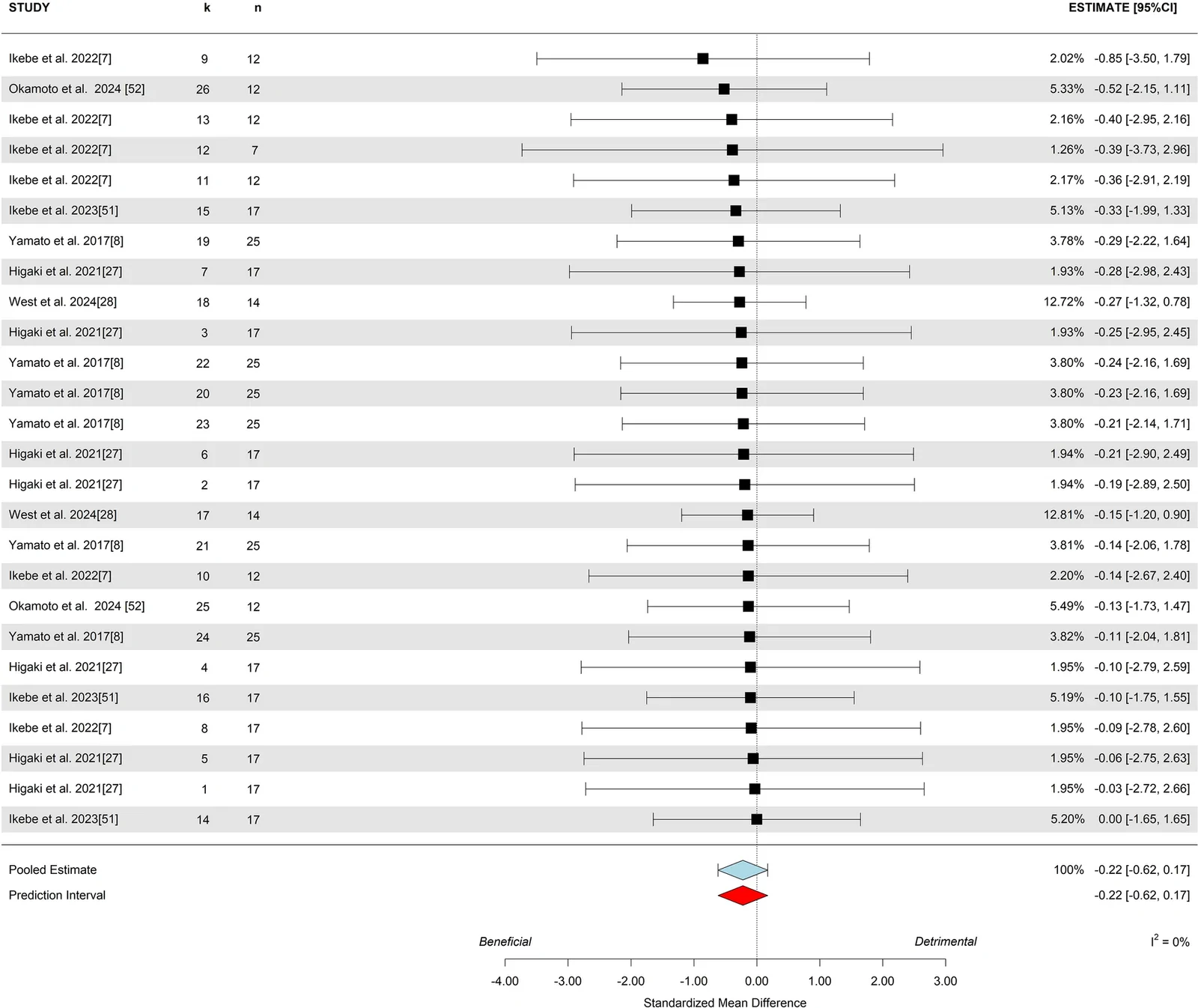
Pooled effect of acute stretching interventions (*u* = 6, *k* = 26, *n* = 110) on pulse wave velocity. Based on the 3-level model, there was a small but significant effect (SMD =  − 0.22, *p* = 0.258). CI, confidence interval; *k*, individual effect estimate; *I*^2^, Higgins’ *I*^2^ (heterogeneity) statistic; SMD, standardized mean difference; *u*, study

The observed SMDs for *k* ranged from − 0.85 (large beneficial effect) to 0 (no effect). The pooled estimates are displayed in the Fig. 2 forest plot. There was a small, beneficial, but nonsignificant pooled effect (SMD =  − 0.22, 95% CI − 0.62 to 0.17, *p* = 0.258), with no change following permutation analysis. The corresponding MD was − 0.14 m/s (95% CI − 0.38 to 0.11, *p* = 0.253). The univariate random-effects estimate was comparable to the multi-level model estimate.

No asymmetry was revealed following inspection of the funnel plot (Supplementary Fig. 2) and regression test (*p* = 0.935). The fail-safe *N* was 0. None of the moderators were significant. No influential *k* or *u* outliers were identified through examination of the Baujat plot or Cook’s distances (Supplementary Fig. 4).

#### Longitudinal Stretching Studies

**Fig. 3:**
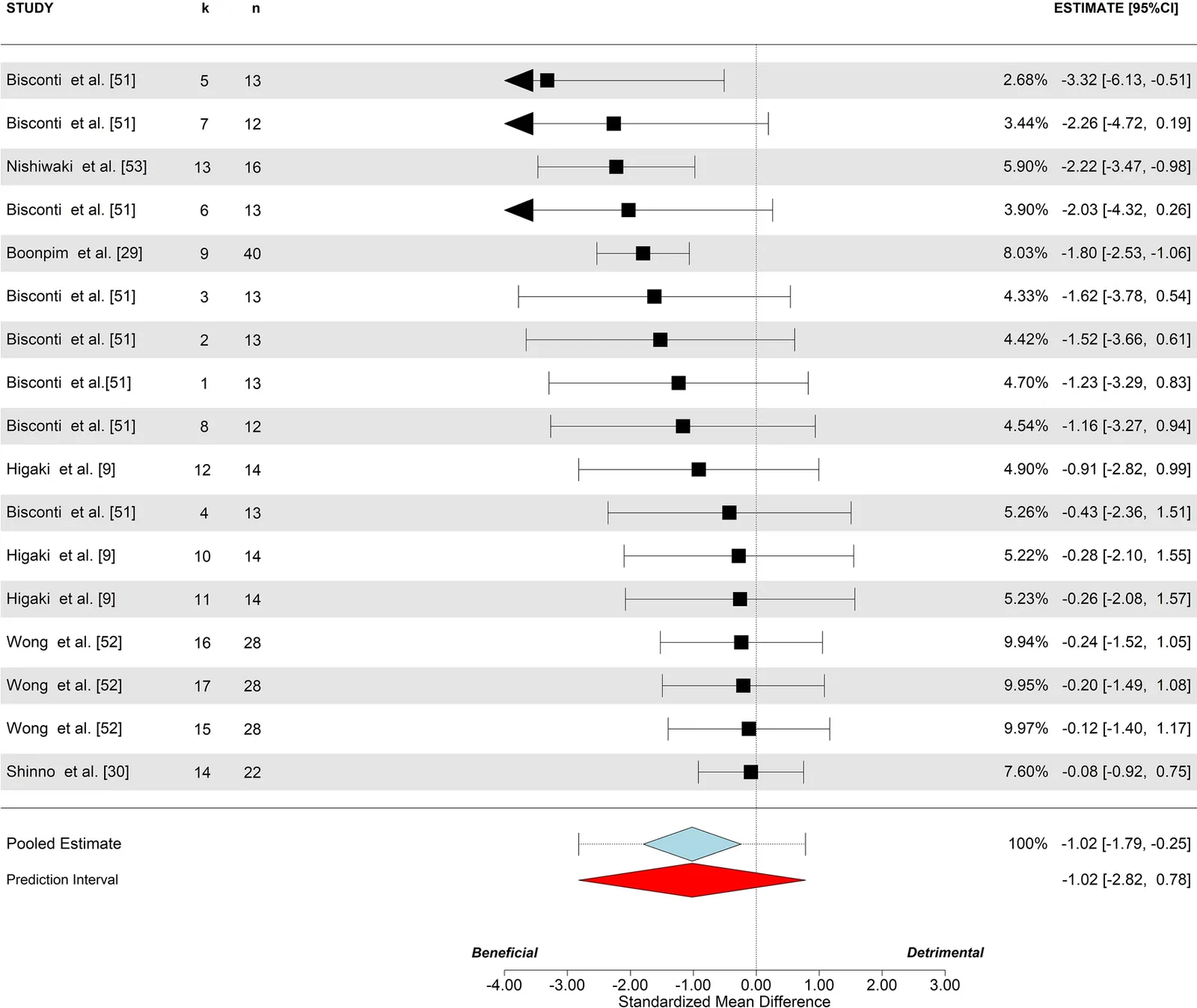
Pooled effect of longitudinal stretching interventions (*u* = 6, *k* = 17, *n* = 159) on pulse wave velocity. Based on the 3-level model, there was a large significant beneficial effect (SMD =  − 1.02, *p* = 0.012). *CI* confidence interval, *k* individual effect estimate, *I*^2^ Higgins’ *I*^2^ (heterogeneity) statistic, *SMD* standardized mean difference, *u* study

All *k* estimates were negative (beneficial), with the SMDs ranging from − 3.32 (large effect) to − 0.08 (trivial effect). The pooled effect estimates are summarized in Table 3 and in the Fig. 3 forest plot. There was a large beneficial (SMD =  − 1.02, 95% CI − 1.79 to − 0.25, *p* = 0.012) pooled effect, with a prediction interval that ranged from − 2.82 to 0.78. The corresponding MD was − 0.94 m/s (95% CI − 1.60 to − 0.28, *p* = 0.013).

**Table 3:** The effects of longitudinal stretching studies

Category	Model	Moderator			Benefit	Contrast				Model fit	
		Variable	Ref	Comparator		SMD	LCI	UCI	*p*	AICc	*τ2*
	0	Intercept-only (no moderators)				− 1.02	− 1.79	− 0.25	0.012	48.4	0.59
Stretching Rx	1	Stretch site	Lower	Upper	N	0.02	− 1.83	1.88	0.979	45.6	0.79
	2	Stretches (#)	Low	High	N	0.63	− 1.56	2.81	0.441	48.7	0.70
	3	Stretch hold (s)	Low	High	Y	− 0.63	− 2.81	1.56	0.550	48.7	0.70
	4	Stretch type	Passive	Active	N	0.63	− 1.56	2.81	0.550	48.7	0.70
	5	Stretch (reps)	Low (< 3)	High (≥ 3)	Y	− 1.46	− 2.23	− 0.70	< 0.001	43.0	0.00
	6	Time (min)	Low (13–29)	High (30–50)	Y	− 0.79	− 1.96	0.38	0.169	48.3	0.47
	7	Sessions (weeks)	Low (3–4)	High (5–7)	N	− 1.05	− 2.52	0.42	0.149	47.8	0.43
Study design	8	Duration (weeks)	Low	High	N	0.31	− 1.50	2.22	0.718	49.4	0.77
	9	Duration (total h)	Low	High	Y	− 0.70	− 2.28	0.87	0.357	49.2	0.55
	10	Supervision	No	Yes	Y	− 1.11	− 2.58	0.37	0.130	47.8	0.42
Study quality	11	Study quality	Low	High	N	0.26	− 1.55	2.07	0.763	49.5	0.78
	12	Risk of bias	n/a								
Outcome	13	Measure	BA	CF	N	− 0.04	− 1.40	1.01	1.325	60.4	0.77
			CR		Y	0.44	− 1.19	1.68	2.057		
			FA		N	− 0.06	− 1.63	1.16	1.522		

Each moderator was independently added to the intercept-only model. The moderator variable along with the reference (Ref.) and comparator is listed. The Benefit column indicates whether the moderator was beneficial (increased the magnitude of change in the outcome) or detrimental

*AICc* small-sample corrected Akaike Information Criterion (lower = better model fit), *LCI* lower confidence interval (95%), *p* significance level, *Pred. Int.* prediction interval, *R2* pseudo coefficient of determination (%variance explained by moderator), *SMD* standardized mean difference, *UCI* upper confidence interval (95%), *τ*^2^ tau squared (model variance), *CF* carotid-femoral PWV, *CR* carotid-radial PWV, *BA* brachial-ankle PWV, *FA* femoral-ankle PWV

No asymmetry was revealed through inspection of the funnel plot (Supplementary Fig. 3) and Egger’s regression test (*p* = 0.386). The fail-safe *N* was 38. Examination of the Baujat plot and Cook’s distances identified 1 k (9) [29] that could be considered influential, but it was not an outlier and, therefore, was not removed (Supplementary Fig. 5). The *k* (9) [29] was likely influential because it had the largest sample size of the included effects. The true outcomes appeared to be heterogeneous (*Q* (16) = 24.1 *p* = 0.088, *I*^2^ = 33.6%, *τ*^2^ = 0.59). Considering *τ*^2^ did not equal zero, a 95% prediction interval was calculated for the pooled SMD; the true outcome was estimated to be between − 2.82 and 0.78. The majority of the variance was at level-3 (51%), with the remainder of the variance at level-1 (49%). In moderation analyses, number of repetitions (intensity) was a significant moderator; three or more repetitions were more effective than fewer repetitions (SMD =  − 1.46, *p* < 0.001). The analysis suggested likely, but not statistically significant, moderating effects of session duration (time), number of sessions per week (frequency), and supervised stretching. Sessions lasting more than 30 min were more effective than those lasting less than 30 min (SMD =  − 0.79, *p* = 0.169) and stretching five or more times per week was more effective than less frequent sessions (SMD =  − 1.05, *p* = 0.149). Additionally, supervised stretching was more effective than unsupervised stretching (SDM =  − 1.11, *p* = 0.130).

## Discussion

### Results Summary

**Fig. 4:**
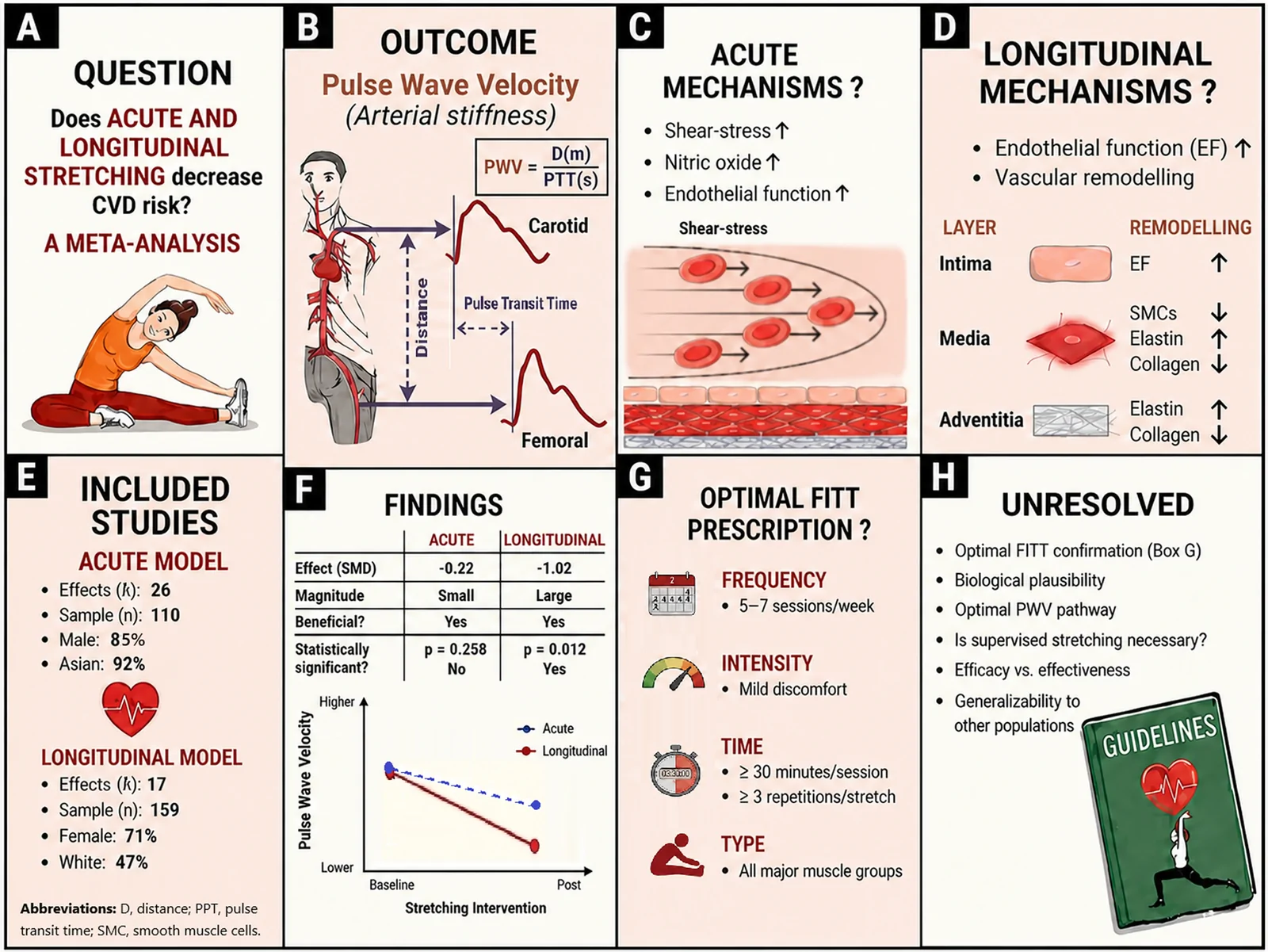
Infographic summarizing meta-analysis **A** question, **B** outcome, **C** acute mechanisms, **D** longitudinal mechanisms, **E** included studies, **F** findings, **G** optimal FITT prescription, and **H** unresolved. **A** This meta-analysis addressed whether acute and longitudinal musculoskeletal stretching interventions decrease cardiovascular disease (CVD) risk in adults aged 18 years or older. **B** The intermediate measure, as a proxy for CVD risk, was pulse wave velocity (PWV). PWV is the speed at which the forward pressure wave is transmitted between a proximal (e.g., carotid) and distal (e.g., femoral) arterial segment, with faster PWV indicative of increased arterial stiffness. **C** A single stretching bout may temporarily decrease arterial stiffness and PWV through acute improvements in endothelial function, plausibly as a function of shear-stress mediated increases in nitric oxide. **D** Repeated acute improvements in endothelial function, resulting from regular exposure to stretching, may lead to extracellular matrix remodeling and a reduction in chronic arterial stiffness. **E** The literature search identified 26 (*k*) for the acute stretching intervention and 17 (*k*) for the longitudinal stretching intervention. The pooled samples were relatively homogeneous, limiting generalizability. **F** Acute stretching led to a small reduction (beneficial) in PWV (SMD =  − 0.22, *p* = 0.258), and longitudinal stretching resulted in a large reduction in PWV (SMD =  − 1.02, *p* = 0.012). **G** Based on the moderation analyses and the current ACSM guidelines, we provisionally recommend that 30–50 min stretching sessions be performed on 5–7 days per week. At each session, the major muscle groups should be stretched to the point of tightness for 10–30 s, three or more times. **H** Unresolved issues include establishing the optimal stretching prescription, clarifying its biological plausibility, identifying the most effective approach to delivering interventions, and enhancing generalizability. *SMCs* smooth muscle cells, *SMD* standardized mean difference

A summary overview is presented in Fig. 4. When summarizing findings from a systematic review, it is important to consider: (1) the magnitude, direction, and precision of the pooled effect; (2) the certainty of the evidence supporting these results; and (3) the clinical significance of the findings. To evaluate the certainty of evidence, we applied the grading of recommendations, assessment, development, and evaluations (GRADE) framework that assesses the domains of imprecision, risk of bias, inconsistency, indirectness, and publication bias.

For the acute stretching interventions, we estimated a small, nonsignificant decrease (beneficial) in PWV (SMD =  − 0.22, *p* = 0.258), with a 95% confidence interval ranging from − 0.62 (moderate, beneficial) to 0.17 (small, harmful). The estimated variance of the observed effect (*τ*^2^) was 0; therefore, a prediction interval was not calculated (no imprecision). As discussed below, the *τ*^2^ value is likely underestimated and may be attributable to sampling variance. Study quality was rated high for each of the 26 k. However, the risk of bias was rated high for 14 k, with some concerns for the remaining 12 k (the risk of bias was downgraded by 1 level). Although the pooled effect did not reach statistical significance, all effect estimates, except 1 k with SMD 0 [51], consistently favored a decrease in PWV (no inconsistency), and each *u* directly measured PWV as an indicator of AS (no indirectness). No publication bias was detected on the basis of funnel plot symmetry (no publication bias). Based on the GRADE framework, evidence was initially rated as high certainty because the included designs incorporated randomized controlled trials. Two studies [8, 27] used contralateral limb controls rather than parallel-group controls. While this design may not meet the strictest definition of a conventional parallel-group RCT, it was considered methodologically rigorous for acute physiological outcomes owing to its ability to minimize inter-individual variability and improve internal validity. This design characteristic was transparently considered within the risk of bias domain. Overall, the certainty of evidence for the acute effects of stretching was rated as moderate, reflecting some concerns about risk of bias.

For the longitudinal stretching interventions, we estimated a large, significant decrease (beneficial) in PWV (SMD =  − 1.02, *p* = 0.012) following a median of 12 weeks of stretching intervention. A 95% confidence interval ranged from − 0.25 (small) to − 1.79 (large). The prediction interval ranged from 0.78 (moderate, detrimental) to − 2.82 (large, beneficial), representing the range of effects that could be observed in future studies (the certainty of evidence was downgraded by one level owing to imprecision). Study quality was rated high for 12 k, but low for the remaining 5 k. There was some risk of bias for all *k* (the certainty of evidence was downgraded by one level owing to risk of bias). All effect estimates uniformly indicated a reduction in PWV (no inconsistency), and all studies directly measured PWV as an indicator of AS (no indirectness). Additionally, the symmetrical funnel plot was observed (no publication bias). Based on the GRADE framework, the certainty of evidence for the longitudinal effects of stretching was rated as low, owing to imprecision and risk of bias. The 0.94 m/s absolute decrease in PWV approaches the value (1 m/s) set to represent clinical significance.

In short, acute stretching interventions resulted in a small, nonsignificant decrease in PWV. A large decrease was found when the stretching intervention was performed longitudinally. Overall, our findings provide preliminary evidence that stretching prescription may be a meaningful intervention for reducing arterial stiffness and potentially lowering CVD risk. The observed findings are tempered by the varying quality of the included studies. Below, we discuss the potential implications of our findings in more detail, including the factors that moderate the observed outcomes.

### Limitations and Strengths

We acknowledge that no study is perfect. Therefore, to provide context and ensure the findings of this review can be fully adjudicated, the known limitations should be considered. First, only a small number of studies (6* u* in the acute stretching intervention meta-analysis [7, 8, 27, 28, 51, 52], and six studies in the longitudinal stretching intervention meta-analysis [9, 29, 30, 53–55]) were included. Regarding sample size considerations, all acute intervention studies employed a cross-sectional design, requiring a minimum of eight subjects per study to have 80% power given high within-subject correlation (*r* =  − 0.99) and a small SMD (− 0.23). All acute intervention studies met or exceeded this sample size threshold. By contrast, longitudinal studies were conducted using RCTs or NRCTs, where a larger sample size of at least 32 subjects was necessary to detect a large SMD (− 1.02) with 80% power. Among these, only one *u* [29] fulfilled the sample size criterion. Furthermore, a priori power calculations were reported in 83% (5 of 6) of acute and 50% (3 of 6) of longitudinal studies. This meta-analysis pooled data (*k*) to increase the likelihood of estimating a robust pooled effect size. However, the robustness of the pooled effect size may be limited by the small number and limited precision of individual* k*—along with the ability to explain between- and within-study variance using moderation analysis. Second, while all the acute stretching studies were of high quality, the longitudinal studies were generally suboptimal, with most lacking information on intervention adherence, except for 1 *u* [54]. Third, for the acute intervention, 2 of the 6 *u* (14/26 k) used the contralateral limb as the control condition [8, 27]. This study design can be an efficient approach for minimizing within-subject variance. However, the measurements made on the control leg may have been biased by the stretching exercises (e.g., increased blood flow and/or sympathetic activity) and/or the measurements made on the experimental leg (e.g., pressor reflex). As such, the true effect may not have been captured. Lastly, the majority of the acute studies (5 out of 6) and longitudinal studies (3 out of 6) were conducted in Japan and recruited only Asian populations [7–9, 27, 29, 30, 51, 52, 55], limiting the generalizability of the findings.

We designed this meta-analysis to ensure the potential limitations were outweighed by known strengths. First, to avoid violating the assumption of *k* independence, we used a three-level model to account for multiple *k* nested within each *u*. The two previous meta-analyses, by Kato et al. in 2020 [25] and Thomas et al. in 2021 [24], either did not account for the nested *k* [25] or completed a separate meta-analysis for each PWV outcome [24]. Second, an additional strength of the three-level meta-analytical approach is the parsing of random variance into that which can (between- and within-study variance) and cannot (sampling variance) be explained, thereby enhancing the strength and interpretability of moderation analysis. Third, we completed independent meta-analyses for acute- and longitudinal-stretching interventions. This approach facilitated insights into the optimal stretching stimulus, as well as its cumulative effects, thereby lending some support towards biological plausibility [20]. Lastly, our meta-analysis pooled data from studies that used various measures/segments of PWV as the outcome and analyzed whether the effect of stretching varies across these PWV measures. PWV is an established noninvasive measure of AS and biological vascular aging [11, 12, 20, 20]. To avoid violating the *k* independence assumption, Thomas et al. [24] analyzed brachial-ankle PWV and carotid-femoral PWV separately, at the cost of statistical power. Similarly, the research question in the study by Kato et al. [25] focused on arterial stiffness*,* and pooled PWV and non-PWV outcomes, including CAVI and augmentation index (AIx).

### Interpretation and Comparison with Other Studies

In this section, we discuss: (1) the magnitude and direction of the effect compared with other studies; (2) unexpected findings; and (3) moderation analysis.

#### Comparison with Other Studies

Both acute and longitudinal stretching interventions decreased (beneficial) PWV, with a small acute decrease (SMD =  − 0.22, *p* = 0.258) and a large decrease (SMD =  − 1.02, *p* = 0.012) following 4 [9] to 12 weeks [30, 53] of prescribed stretching. Our findings are consistent with the previous meta-analyses completed by Kato et al. in 2020 [25] and Thomas et al. in 2021 [24]. Kato et al. [25] similarly reported a large decrease (SMD − 1.00, 95% CI − 1.57 to − 0.44, *p* < 0.001) following 4–12 weeks of stretching intervention. However, the comparability to our study is at least partially limited by Kato et al.’s inclusion of non-PWV measures (CAVI) and a measure (AIx) that does not reflect AS. Thomas et al. [24] reported a large decrease in brachial-ankle PWV (SMD − 2.04, 95% CI − 3.06 to − 1.01) and a moderate decrease in carotid-femoral PWV (SMD − 0.46, 95% CI − 0.67 to − 0.24). The comparability to our study may be somewhat limited by Thomas et al.’s inclusion of acute and longitudinal stretching interventions in the same meta-analysis model. While the previous meta-analyses do have some limitations, as does the current meta-analysis (see above section), the reported findings are complementary and provide additional preliminary evidence to support reductions in AS as a result of stretching (especially longitudinal) interventions.

#### Unexpected Findings

The random variance (*τ*^2^) for the acute stretching meta-analysis was zero, implying that our pooled estimate reflects one underlying effect. This is unlikely considering the included studies differed in terms of study design, stretching prescription, and selected outcome measures. The *τ*^2^ value is likely underestimated owing to sampling variance. A closer inspection of the CIs across effects (*k*) supports this notion. The pooled effect CI includes zero (− 0.62 to 0.17), and the individual effect CIs are wide, indicating limited effect precision. This is potentially attributable to the small sample sizes across *k* (median of 17 for acute stretching [7, 8, 27, 28, 51, 52]). Additionally, while study quality was rated high for each of the 26 k, the risk of bias was rated high for 14 k, with some concerns for the remaining 12 k*.*

#### Moderation Analysis

The longitudinal stretching meta-analysis pooled effect was moderated by stretching prescription. The American College of Sports Medicine’s (ACSM’s) guidelines, based on the FITT principle, recommend that on 2–3 days per week (frequency) each major muscle–tendon unit (type) is stretched to the point of tightness (intensity) for 10–30 s (time) [26]. However, these guidelines are designed to improve flexibility, not to decrease CVD risk. We observed a greater effect size magnitude when each stretch was repeated three or more times (SMD contrast =  − 1.46). Despite the absence of statistical significance, the effect estimates consistently favored a reduction in PWV when each session lasted 30–50 min versus 13–29 min (SMD contrast =  − 0.79), and when the frequency of stretching was 5–7 days per week versus 3–4 days per week (SMD contrast =  − 1.05). Stretching sites, number of stretching sites, stretch holding time, and stretching type (active versus passive) did not moderate the pooled effect. While additional research is warranted to test and fine-tune the optimal prescription for CVD risk reduction, our findings are generally in accord with the ACSM guidelines for improving flexibility. Based on the available evidence, stretching may provide vascular benefits, and we can provisionally recommend that 30–50 min stretching sessions (time) are performed on most days (5–7) of the week (frequency). While our findings for stretch type are null, we recommend that each muscle–tendon group (type) is stretched to the point of tightness (intensity) for 10–30 s and repeated three or more times (time).

With respect to study design, the effect size magnitude trended toward being greater when the stretching sessions were supervised (SMD contrast =  − 1.11). A logical next step is an efficacy study, to confirm the magnitude of PWV reduction under controlled conditions, including supervision of stretching sessions. This step would better establish clinical significance and set a baseline for future interventions. That said, confirming efficacy does not mean an intervention will be effective. For example, while supervising stretching sessions can improve adherence and intervention quality, it will also decrease feasibility and scalability. Stretching intervention could be an attractive option for mitigating CVD risk because it requires minimal skill and can be performed in most locations, making it accessible and sustainable for a wide range of populations [5, 6]. Once efficacy is confirmed, effectiveness trials will be required to test how well stretching interventions perform under real-world conditions, and implementation research will be critical to elucidate strategies that will enhance adoption, feasibility, adherence, and scalability.

The study outcome method (i.e., carotid-femoral PWV, brachial-ankle PWV, carotid-radial PWV, and femoral-ankle PWV) did not moderate the pooled effect for either the acute or longitudinal interventions. These findings, though supported by a limited number of studies, imply that stretching interventions may similarly decrease (improve) peripheral and central arterial stiffness. Additional research is required to further our understanding of the effects of stretching across the whole vascular tree. Our recommendation is that future studies use as the primary outcome a measure of central PWV, such as carotid-femoral PWV, brachial-femoral PWV, or heart-femoral PWV. This is because central PWV is more strongly linked to CVD [11, 12], normative data are available [11, 12], and using a more consistent approach will improve comparison between studies.

### Clinical and Research Significance and Implications

In this section, we discuss the clinical significance, covering potential benefits and harms, as well as the implications, including recommendations for future research (see Table 4 and Fig. 4).

**Table 4:** Summary of implications arising from meta-analysis completion

What did we know prior to this study?
1 m/s ↑ PWV = ↑ CVD event risk by 14% 2 × meta-analyses reported stretching = ↓ PWV (beneficial)
What did we not know prior to this study?
Independent effect of acute and longitudinal stretching Optimal stretching prescription Importance of effect moderators
What does this study add?
Acute stretching = small ↓ PWV (standardized mean difference [SMD] = − 0.22, *p* = 0.258) Longitudinal stretching = large ↓ PWV (SMD = − 1.02, *p* = 0.012) Optimal prescription: 3 reps/stretch, 30–50 min/session, 5-7 sessions/week, supervised
How do we use this new information?
Stretching interventions = potentially ↓ CVD risk Moderator analysis findings = future intervention design
What needs to happen next to move the field forward?
Efficacy: randomized controlled trials to establish clinical significance Effectiveness: stretching intervention in real-world and unsupervised conditions Implementation: adoption, feasibility, adherence, and scalability Stretching prescription: confirm/optimize Generalizability: age, sex, race/ethnicity, and body composition

*PWV* pulse wave velocity, *CVD* cardiovascular disease, *SMD* standardized mean difference, *min* minutes, *reps* repetitions

Acute stretching intervention resulted in a small (SMD − 0.22), nonsignificant decrease (beneficial) in PWV, whereas longitudinal interventions led to a large, (SMD − 1.02) significant decrease. While additional research is required to more robustly establish biological plausibility [20], our findings suggest that the benefits of stretching are cumulative. The 0.94 m/s absolute decrease in PWV, which approaches the value (1 m/s) set to signify clinical significance, compares favorably to the − 0.67 m/s (95% CI − 0.97 to − 0.38) decrease estimated by a meta-analysis of 26 aerobic exercise RCTs [56]. While the quality of evidence supporting aerobic exercise-induced reductions in PWV is more robust than what is available for stretching interventions—potentially making direct comparison misleading—the preliminary findings are promising. Further encouragement comes from the absence of reported adverse effects in the stretching interventions included in the meta-analyses. Additionally, all six longitudinal stretching interventions reported high adherence. Only Wong et al. [54] noted drop-outs; 2 out of 17 participants withdrew, citing personal reasons unrelated to the intervention.

In the previous section, we made preliminary recommendations on the basis of the FITT principle for stretching prescription. However, owing to the imprecision and risk of bias across the longitudinal intervention, and the small, homogenous samples of existing studies, the overall certainty of evidence remains limited. Rigorous efficacy trials (e.g., RCT) are required to test and fine-tune the prescription and establish a biologically plausible relationship between stretching, PWV reduction, and CVD risk. Importantly, because carotid-femoral PWV is the gold standard measure of arterial stiffness and a well-established predictor of CVD risk, future studies should prioritize carotid-femoral PWV as the primary outcome rather than alternative indices such as brachial-ankle PWV. This will also ensure greater consistency and comparability between studies. Following efficacy trial(s), effectiveness and implementation research are needed to maximize the likelihood of stretching interventions working under real-world conditions. Finally, there also remains a need to extend generalizability and test whether the stretching prescription should be tailored to different population segments. In particular, factors such as age, sex, race/ethnicity, and disease status are likely to influence vascular responses to stretching interventions. For example, postmenopausal women may experience distinct changes in cardiovascular function due to the loss of estrogen, and age–sex interactions have been shown to affect adaptations to various forms of exercise. These considerations, along with the limitations of the current evidence base, highlight the importance of future studies that stratify participants by demographic and clinical characteristics to better understand for whom stretching interventions are most effective.

## Conclusions

The objective of this meta-analysis was to test whether musculoskeletal stretching interventions decrease (beneficial) PWV. PWV is a vital biomarker for noninvasive measurement of arterial stiffness and CVD risk assessment [12]. We found that a single bout of stretching resulted in a small decrease in PWV (SMD =  − 0.22, 95% CI − 0.62 to 0.17), with 4–12 weeks of regularly prescribed stretching leading to a large decrease in PWV (SMD =  − 1.02, 95% CI − 1.79 to − 0.25). The pooled effects compare favorably with the effect observed from aerobic exercise interventions. Collectively, our findings indicate that stretching interventions may be an attractive strategy to mitigate arterial stiffness. We provisionally recommend that 30–50 min (time) stretching sessions are performed on most days (5–7) of the week (frequency). At each session, the major muscle–tendon groups (type) should be stretched to the point of tightness (intensity) for 10–30 s and repeated three or more times (time). The findings from this meta-analysis may be used to help design future RCTs to test the efficacy and biological plausibility of musculoskeletal stretching interventions in reducing arterial stiffness and, potentially, CVD risk.

## Supplementary Information

Below is the link to the electronic supplementary material.

Supplementary file1 (DOCX 3437 kb)

## Declarations

### Data availability

The data analyzed for this meta-analysis and the corresponding R script are available from the corresponding author on reasonable request.
