# Supplementary material for: Musculoskeletal Stretching and Arterial Stiffness: Systematic Review and Meta-analysis of Acute and Longitudinal Interventions
Source: Sports Med. 2026 Jul 26;56(11):2823–40. doi: 10.1007/s40279-026-02483-8 (PMC13672256; doi:10.1007/s40279-026-02483-8)
Supplement: Supplementary file 1 [file 40279_2026_2483_MOESM1_ESM.docx]

SUPPLEMENT

**MANUSCRIPT TITLE:** Musculoskeletal Stretching and Arterial Stiffness: Systematic Review and Meta-Analysis of Acute and Longitudinal Interventions

**SHORT TITLE:** Stretching and Arterial Stiffness

**CONTENTS**

[INTRODUCTION TO THE SUPPLEMENT	2](#_Toc216792290)

[TABLE S1.	3](#_Toc216792291)

[Title: Definition of terms used in the supplement.	3](#_Toc216792292)

[TABLE S2	4](#_Toc216792293)

[Title: Search strategy………………………………………………………………………………………………………………………………………………………………………… 4](#_Toc216792294)

[TABLE S3	4](#_Toc216792293)

[Title: Intervention details of acute stretching studies.	4](#_Toc216792294)

[TABLE S4	5](#_Toc216792295)

[Title: Intervention details of longitudinal stretching studies.	5](#_Toc216792296)

[FIGURE S1.	6](#_Toc216792297)

[Title: Cochrane Risk-of-Bias 2 (ROB2)	6](#_Toc216792298)

[FIGURE S2.	7](#_Toc216792299)

[Title: Funnel plot for acute stretching studies.	7](#_Toc216792300)

[FIGURE S3	8](#_Toc216792301)

[Title: Funnel plot for longitudinal stretching studies	8](#_Toc216792302)

[FIGURE S4	9](#_Toc216792303)

[Title: Baujat plot for acute stretching studies.	9](#_Toc216792304)

[FIGURE S5	10](#_Toc216792305)

[Title: Baujat plot for longitudinal stretching studies.	10](#_Toc216792306)

[REFERENCES	11](#_Toc216792307)

# INTRODUCTION TO THE SUPPLEMENT

This supplement provides the following additional information:

1. Search strategy in PubMed
2. Intervention details for each effect (*k*)
3. Risk of Bias scoring
4. Meta-analysis assumptions

To aid in interpreting the material presented below, the following table contains explanations of the abbreviations and terms used.

# TABLE S1.

## Title: Definition of terms used in the supplement.

|  | **Term/Subject** | **Abbreviation** | **Interpretation** | **Table** | **Fig** |
| --- | --- | --- | --- | --- | --- |
| 1. Intervention | Study | *u* | Individual article/study. | 1-2 |  |
|  | Effect | *k* | Unit included in the meta-analysis. | 1-2 |  |
|  | Measure post-stretch (min) | n/a | Duration between last stretch vs. PWV measurement. | 1-2 |  |
|  | Mono or Bi [limb(s)] | n/a | One versus both limbs. | 1-2 |  |
|  | Active/Passive | n/a | Active stretching versus passive stretching | 1-2 |  |
|  | Stretch Site | n/a | Stretched body sites | 1-2 |  |
|  | Hold (sec) | n/a | Duration of time a single stretch is held. | 1-2 |  |
|  | Relax (sec) | n/a | Duration of rest time between stretches. | 1-2 |  |
|  | Repetitions | Reps (#) | # each stretch is repeated. | 1-2 |  |
|  | Stretch exercise (#) | n/a | # stretching exercises. | 1-2 |  |
|  | Minutes per session | Min/Sessions | Duration of each stretching exercise session. | 1-2 |  |
|  | Total hours | n/a | Total hours of stretching across intervention. | 2 |  |
|  | Sessions per week | Sessions/wk | # stretching sessions per week. | 2 |  |
|  | Weeks | Wks | Duration of intervention. | 2 |  |
|  | Cross-over trial | X-Over | Cross-over trial design. |  | 1-2 |
|  | Randomized controlled trial | RCT | Randomized controlled trial design. |  | 1-2 |
| 2. Bias | Risk-Of-Bias 2 | ROB2 | Risk of Bias assessment tool. |  | 1-2 |
|  | Dimension 1 | D1 | Bias: randomization process. |  | 1-2 |
|  | Dimension 2 | D2 | Bias: period and carryover effects. |  | 1-2 |
|  | Dimension 3 | D3 | Bias: deviations to intended intervention. |  | 1-2 |
|  | Dimension 4 | D4 | Bias: missing outcome data. |  | 1-2 |
|  | Dimension 5 | D5 | Bias: outcome measurement. |  | 1-2 |
|  | Overall | n/a | Overall bias. |  | 1-2 |
|  | Low risk | n/a | Low risk of bias. |  | 1-2 |
|  | Some Concerns | n/a | Some concerns about bias. |  | 1-2 |
|  | High Risk | n/a | High risk of bias. |  | 1-2 |
| 3. Meta | Funnel Plot | n/a | No publication bias = pyramid shaped. |  | 2-3 |
|  | Baujat Plot | n/a | Values in top right corner = influential. |  | 4-5 |
|  | Cook’s D / Cook’s Critical | n/a | Influential observation = Cook’s Critical value exceeded. |  | 4-5 |
|  | Influence | n/a | Impacts of individual study on pooled effects. |  | 4-5 |
|  | Heterogeneity | n/a | Variability in effect sizes across studies. |  | 4-5 |

INTERPRETATION: A full definition of terms

# TABLE S2.

## Title: Search strategy.

| **Database:** [www.pubmed.gov](http://www.pubmed.gov/) | | |  |
| --- | --- | --- | --- |
| **Search date:** Dec 6, 2025 | | |  |
| **Limits:** Humans; English language; Date from 1974 | | |  |
|  |  |  |  |
| **Set#** | **Description** | **Terms** | **Result (count)** |
| **1** | **Outcome terms** | “arterial stiffness” OR “pulse wave velocity” OR PWV OR “vascular stiffness”  Translations: "arterial stiffness"[All Fields] OR "pulse wave velocity"[All Fields] OR "PWV"[All Fields] OR "vascular stiffness"[All Fields] |  |
| **2** | **Intervention terms** | Stretching  Translations: "stretch"[All Fields] OR "stretched"[All Fields] OR "stretches"[All Fields] OR "stretching"[All Fields] OR "stretchings"[All Fields] |  |
| **3** | **Population terms** |  |  |
| **4** | **Comparison** |  |  |
| **5** | **Study type terms** |  |  |
| **6** | **Combine above searches** | #1 AND #2 | 140 |
| **7** | **Restrict to English language articles** | #6 AND English[lang] | 138 |
| **8** | **Exclude animal articles** | #7 NOT (animals[mh]) | 76 |

# TABLE S3

## Title: Intervention details of acute stretching studies.

| Author  Year [Ref] | *u* | *k* | Measure  Post-stretch (min) | Stretch  Site | Mono  or Bi | Active/  Passive | Hold  (sec) | Relax  (sec) | Reps. (#) | Stretch  exercises (#) | Min/ session |
| --- | --- | --- | --- | --- | --- | --- | --- | --- | --- | --- | --- |
| Higaki *et al*. 2021 [1] | 1 | 1 | 1 | Leg | Mono | Passive | 30 | 5 | 6 | 1 | 3.50 |
| Higaki *et al*. 2021 [1] | 1 | 2 | 1 | Leg | Mono | Passive | 30 | 10 | 6 | 1 | 4.00 |
| Higaki *et al.* 2021 [1] | 1 | 3 | 1 | Leg | Mono | Passive | 30 | 20 | 6 | 1 | 5.00 |
| Higaki *et al.* 2021 [1] | 1 | 4 | 1 | Leg | Mono | Passive | 30 | 60 | 6 | 1 | 9.00 |
| Higaki *et al.* 2021 [1] | 1 | 5 | 1 | Leg | Mono | Passive | 30 | 5 | 6 | 1 | 3.50 |
| Higaki *et al.* 2021 [1] | 1 | 6 | 1 | Leg | Mono | Passive | 30 | 10 | 6 | 1 | 4.00 |
| Higaki *et al.* 2021 [1] | 1 | 7 | 1 | Leg | Mono | Passive | 30 | 20 | 6 | 1 | 5.00 |
| Higaki *et al.* 2021 [1] | 1 | 8 | 1 | Leg | Mono | Passive | 30 | 60 | 6 | 1 | 9.00 |
| Ikebe *et al.* 2022 [2] | 2 | 9 | 5 | Trunk | Bi | Passive | 30 | 30 | 5 | 6 | 30.00 |
| Ikebe *et al.* 2022 [2] | 2 | 10 | 5 | Trunk | Bi | Passive | 30 | 30 | 5 | 6 | 30.00 |
| Ikebe *et al.* 2022 [2] | 2 | 11 | 5 | Trunk | Bi | Passive | 30 | 30 | 5 | 6 | 30.00 |
| Ikebe *et al.* 2022 [2] | 2 | 12 | 5 | Leg | Mono | Passive | 30 | 10 | 6 | 1 | 4.00 |
| Ikebe *et al.* 2022 [2] | 2 | 13 | 5 | Leg | Mono | Passive | 45 | 15 | 5 | 4 | 20.00 |
| Ikebe *et al.* 2023 [3] | 3 | 14 | 0 | Cervical | Bi | Active | 30 | 10 | 2 | 10 | 13.33 |
| Ikebe *et al.* 2023 [3] | 3 | 15 | 15 | Cervical | Bi | Active | 30 | 10 | 2 | 10 | 13.33 |
| Ikebe *et al.* 2023 [3] | 3 | 16 | 30 | Cervical | Bi | Active | 30 | 10 | 2 | 10 | 13.33 |
| West *et al.* 2024 [4] | 4 | 18 | 10 | Trunk | Bi | Passive | 30 | 30 | 5 | 6 | 30.00 |
| West *et al.* 2024 [4] | 4 | 17 | 10 | Trunk | Bi | Passive | 30 | 30 | 5 | 6 | 30.00 |
| Yamato et al. 2017 [5] | 5 | 19 | 0 | Leg | Mono | Passive | 30 | 10 | 6 | 1 | 4.00 |
| Yamato *et al.* 2017 [5] | 5 | 20 | 15 | Leg | Mono | Passive | 30 | 10 | 6 | 1 | 4.00 |
| Yamato *et al.* 2017 [5] | 5 | 21 | 30 | Leg | Mono | Passive | 30 | 10 | 6 | 1 | 4.00 |
| Yamato *et al.* 2017 [5] | 5 | 22 | 0 | Leg | Mono | Passive | 30 | 10 | 6 | 1 | 4.00 |
| Yamato *et al.* 2017 [5] | 5 | 23 | 15 | Leg | Mono | Passive | 30 | 10 | 6 | 1 | 4.00 |
| Yamato *et al.* 2017 [5] | 5 | 24 | 30 | Leg | Mono | Passive | 30 | 10 | 6 | 1 | 4.00 |
| Okamoto *et al.* 2024 [6] | 6 | 25 | 0 | Whole | Bi | Active | 30 | 10 | 3 | 11 | 30.00 |
| Okamoto *et al.* 2024 [6] | 6 | 26 | 30 | Whole | Bi | Active | 30 | 10 | 3 | 11 | 30.00 |

# TABLE S4

## Title: Intervention details of longitudinal stretching studies.

| Author Year | *u* | *k* | Measure Post-stretch  (min) | Stretch Site | Mono or Bi | Active/  passive | Hold (sec) | Relax (sec) | Reps  (#) | Stretch  exercises (#) | Min/  session | T[otal](http://total.hr/)  [hour](http://total.hr/)s | Sessions/ wk | Wks |
| --- | --- | --- | --- | --- | --- | --- | --- | --- | --- | --- | --- | --- | --- | --- |
| Bisconti *et al.* 2020 [7] | 1 | 1 | 20 | Legs | Bi | Passive | 45 | 15 | 5 | 8 | 40.0 | 40.0 | 5.0 | 12.0 |
| Bisconti *et al.* 2020 [7] | 1 | 2 | 20 | Legs | Bi | Passive | 45 | 15 | 5 | 8 | 40.0 | 40.0 | 5.0 | 12.0 |
| Bisconti *et al.* 2020 [7] | 1 | 3 | 20 | Leg | Mono | Passive | 45 | 15 | 5 | 4 | 20.0 | 20.0 | 5.0 | 12.0 |
| Bisconti *et al.* 2020 [7] | 1 | 4 | 20 | Leg | Mono | Passive | 45 | 15 | 5 | 4 | 20.0 | 20.0 | 5.0 | 12.0 |
| Bisconti *et al.* 2020 [7] | 1 | 5 | 20 | Legs | Bi | Passive | 45 | 15 | 5 | 8 | 40.0 | 40.0 | 5.0 | 12.0 |
| Bisconti *et al.* 2020 [7] | 1 | 6 | 20 | Legs | Bi | Passive | 45 | 15 | 5 | 8 | 40.0 | 40.0 | 5.0 | 12.0 |
| Bisconti *et al.* 2020 [7] | 1 | 7 | 20 | Leg | Mono | Passive | 45 | 15 | 5 | 4 | 20.0 | 20.0 | 5.0 | 12.0 |
| Bisconti *et al.* 2020 [7] | 1 | 8 | 20 | Leg | Mono | Passive | 45 | 15 | 5 | 4 | 20.0 | 20.0 | 5.0 | 12.0 |
| Boonpim *et al.* 2017 [8] | 2 | 9 | 10 | Whole | Bi | Active | 20 | 40 | 3 | 10 | 30.0 | 15.0 | 5.0 | 6.0 |
| Higaki *et al.* 2022 [9] | 3 | 10 | NR | Legs | Bi | Active | 30 | 10 | 2 | 10 | 13.3 | 3.6 | 4.0 | 4.0 |
| Higaki *et al.* 2022 [9] | 3 | 11 | NR | Legs | Bi | Active | 30 | 10 | 2 | 10 | 13.3 | 3.6 | 4.0 | 4.0 |
| Higaki *et al.* 2022 [9] | 3 | 12 | NR | Legs | Bi | Active | 30 | 10 | 2 | 10 | 13.3 | 3.6 | 4.0 | 4.0 |
| Nishiwaki *et al.* 2015 [10] | 4 | 13 | 15 | Whole | Bi | Active | 20 | 35 | 3 | 11 | 30.3 | 10.1 | 5.0 | 4.0 |
| Shinno *et al.* 2017 [11] | 5 | 14 | 10 | Whole | Bi | Active | 25 | ? | ? | 15 | 15.00 | 21.0 | 7.0 | 12.0 |
| Wong *et al.* 2014 [12] | 6 | 15 | 50 | Whole | Bi | Both | 30 | 15 | 2 | 38 | 50.0 | 20.0 | 3.0 | 8.0 |
| Wong *et al.* 2014 [12] | 6 | 16 | 50 | Whole | Bi | Both | 30 | 15 | 2 | 38 | 50.0 | 20.0 | 3.0 | 8.0 |
| Wong *et al.* 2014 [12] | 6 | 17 | 50 | Whole | Bi | Both | 30 | 15 | 2 | 38 | 50.0 | 20.0 | 3.0 | 8.0 |

# FIGURE S1.

## Title: Cochrane Risk-of-Bias 2 (ROB2)

# FIGURE S2.

## Title: Funnel plot for acute stretching studies.

# FIGURE S3

## Title: Funnel plot for longitudinal stretching studies

# FIGURE S4

## Title: Baujat plot for acute stretching studies.

# FIGURE S5

## Title: Baujat plot for longitudinal stretching studies.

# REFERENCES

1. Higaki Y, Yamato Y, Fujie S, Inoue K, Shimomura M, Kato S, et al. Acute effects of the different relaxation periods during passive intermittent static stretching on arterial stiffness. PLOS ONE. Public Library of Science; 2021;16:e0259444. https://doi.org/10.1371/journal.pone.0259444

2. Ikebe H, Takiuchi S, Oi N, Takayanagi Y, Makino A, Itoh M, et al. Effects of trunk stretching using an exercise ball on central arterial stiffness and carotid arterial compliance. Eur J Appl Physiol. 2022;122:1205–16. https://doi.org/10.1007/s00421-022-04912-8

3. Ikebe H, Oi N, Makino A, Kume D, Ishido M, Nakamura T, et al. Effects of acute cervical stretching on arterial wall elastic properties. Front Physiol. 2023;14:1198152. https://doi.org/10.3389/fphys.2023.1198152

4. West K, Spears K, Joyner B, Smith C, Jenkins NDM, Grosicki GJ, et al. The acute effect of passively assisted trunk stretching on central arterial stiffness and blood pressure in middle-aged to older adults. Eur J Appl Physiol. 2024; https://doi.org/10.1007/s00421-023-05389-9

5. Yamato Y, Hasegawa N, Fujie S, Ogoh S, Iemitsu M. Acute effect of stretching one leg on regional arterial stiffness in young men. Eur J Appl Physiol. 2017;117:1227–32. https://doi.org/10.1007/s00421-017-3609-x

6. Okamoto T, Hashimoto Y, Iemitsu M, Ogoh S. Acute effects of static stretching exercise-induced decrease in arterial stiffness on maximal aerobic capacity. J Sports Med Phys Fitness. 2024;64:849–56. https://doi.org/10.23736/S0022-4707.24.15758-1

7. Bisconti AV, Cè E, Longo S, Venturelli M, Coratella G, Limonta E, et al. Evidence for improved systemic and local vascular function after long-term passive static stretching training of the musculoskeletal system. J Physiol. 2020;598:3645–66. https://doi.org/10.1113/JP279866

8. Boonpim H, Wanpen S, Chanavirut R, Apaijit K, Kukongviriyapan U, Nakmareong S. Reduced Arterial Stiffness and Ankle Blood Pressure Following Stretching Exercise in Postmenopausal Women. Journal of Physiological and Biomedical Sciences. 2017;30:47–51.

9. Higaki Y, Fujie S, Yamato Y, Oshiden M, Iemitsu M. Four weeks of lower-limb static stretching reduces regional arterial stiffness in middle-aged and older women. Phys Act Nutr. 2022;26:022–7. https://doi.org/10.20463/pan.2022.0010

10. Nishiwaki M, Yonemura H, Kurobe K, Matsumoto N. Four weeks of regular static stretching reduces arterial stiffness in middle-aged men. Springerplus. 2015;4:555. https://doi.org/10.1186/s40064-015-1337-4

11. Shinno H, Kurose S, Yamanaka Y, Higurashi K, Fukushima Y, Tsutsumi H, et al. Evaluation of a static stretching intervention on vascular endothelial function and arterial stiffness. Eur J Sport Sci. 2017;17:586–92. https://doi.org/10.1080/17461391.2017.1284267

12. Wong A, Figueroa A. Eight weeks of stretching training reduces aortic wave reflection magnitude and blood pressure in obese postmenopausal women. J Hum Hypertens. 2014;28:246–50. https://doi.org/10.1038/jhh.2013.98
